# The impact of videolaparoscopic surgery in the treatment of endometriosis on depression levels

**DOI:** 10.1590/1806-9282.20231651

**Published:** 2024-08-16

**Authors:** João Nogueira, Vinicius Gonçalves Melo, Luna Carolina Silva Lima, Ana Beatriz Coelho Mendes, Fernanda Nogueira Barreto, Lyvia Maria Rodrigues de Sousa Gomes, Plinio da Cunha Leal

**Affiliations:** 1 Universidade Federal do Maranhão, College of Medicine, Department of Medicine - São Luís (MA), Brazil.; 2 Hospital São Domingos, Gynecology Service, São Luís (MA), Brazil.

**Keywords:** Endometriosis, Depressive symptoms, Surgical procedures, Operative

## Abstract

**OBJECTIVE:**

The aim of the study was to evaluate the impact of laparoscopic surgical treatment of endometriosis on the levels of health-related depression in patients using a validated questionnaire.

**METHODS:**

A prospective study was carried out between September 2020 and May 2022 in a private hospital (São Luís, Maranhão, Brazil), which analysed depression using the Beck Depression Inventory-II, on 103 patients undergoing surgical treatment for endometriosis, evaluated preoperatively and 3 and 6 months after the procedure. Patients with unsuccessful clinical treatment for endometriosis and pain level ≥7 on Visual Analog Scale and who agreed to participate in the study were included. Demographic data were acquired by consulting medical records.

**RESULTS:**

The average age of the participants was 36±6.3 years; the majority of patients were brown (68.6%), married (66.6%), overweight (55.8%), had had hormonal treatments with progestogens (50.9%), low fertility (50.9%), severe endometriosis (39.3%), endometriosis surgery+myomectomy (29.4%) and one (1%) patient withdrew from the study. There was a statistically significant reduction in mean Beck Depression Inventory between the preoperative period and 6 months after surgery (p<0.0001).

**CONCLUSION:**

Surgical treatment of endometriosis appears to have a positive impact on the symptoms of depression in the patients evaluated.

## INTRODUCTION

Endometriosis is an oestrogen-dependent chronic inflammatory condition characterised by the presence of the glands and stroma of endometrial-like tissue outside the uterine cavity, and the condition affects an average of 10% of women of reproductive age^1–3^.

A characteristic of the disease is the delayed diagnosis, which varies according to country and study group and ranges from approximately 4 to 10 years from symptom onset^1,4^. The gold standard method of diagnosis of endometriosis is videolaparoscopy, but in recent years, imaging tests have been implemented and are widely used^3,5^.

Endometriosis usually causes pain and infertility with reduced quality of life, sexual disorder, bipolar disorder, anxiety and depression, in addition to being related to chronic pelvic pain and the occurrence of alexithymia, somatisation, low self-esteem and pain catastrophising^6–8^. Individuals with chronic debilitating diseases have a higher prevalence of depressive symptoms, and this comorbidity can have harmful effects on the clinical condition of the patient^9^.

Studies show that patients with endometriosis have more depressive states than patients without this pathology^5,7,10,11^. The impact of pain in the population with endometriosis is individualised and does not depend on the stage of the disease, suggesting that it is the intensity of the pain that leads to psychological distress and its consequences, not the endometriosis itself^10,11^. One study showed that psychological distress leads to pain catastrophising and predisposes patients to think repeatedly and amplify negative thoughts, which leads to an impact on mental health and pain intensity^11^. Catastrophising negatively influences depression and hinders responses to treatments, making early investigation and detection of this disorder essential for adequate treatment and cost reduction^11^.

The treatment of endometriosis is clinical and/or surgical. Initially, clinical treatment is usually empirical with menstrual cycle blockade, and when this is not successful, surgical treatment may be indicated^1,2^. Surgical treatment also proceeds in cases of suspected ovarian cancer and intestinal or urinary tract obstructions^1,2^. The treatment involves a multidisciplinary team, including physical therapists, psychologists, nutritionists, and sometimes psychiatrists^1^.

The search for solutions to improve depression in patients with endometriosis is essential to benefit these women. In this sense, this study aims to identify whether surgical treatment of endometriosis has an impact on improving the depressive state of these patients and evaluate its impact.

## METHODS

This is a prospective cohort study, carried out on patients with an indication for surgical treatment for endometriosis that was performed by the gynaecological surgery team at Hospital São Domingos, São Luís, Maranhão, Brazil, from September 2020 to May 2022. Endometriosis was suspected based on clinical data and/or suggestive imaging tests. Demographic data were acquired by consulting medical records.

Symptomatic patients with clinical treatment for pain attributed to endometriosis, who had unsuccessful clinical hormonal treatment for at least 3 months and with pain level ≥7 according to the Visual Analog Scale (VAS) were included.

Patients with previous endometriosis surgeries, cancer diagnosis, major surgical complications, asymptomatic cases of endometriosis with surgical indication, pain level ≥7 on VAS ≥7, those who initially did not want to participate in the study and patients with chronic pelvic pain according to the American College of Obstetricians and Gynecologists’ Committee on Practice Bulletins^12^ were excluded.

**Figure 1:**
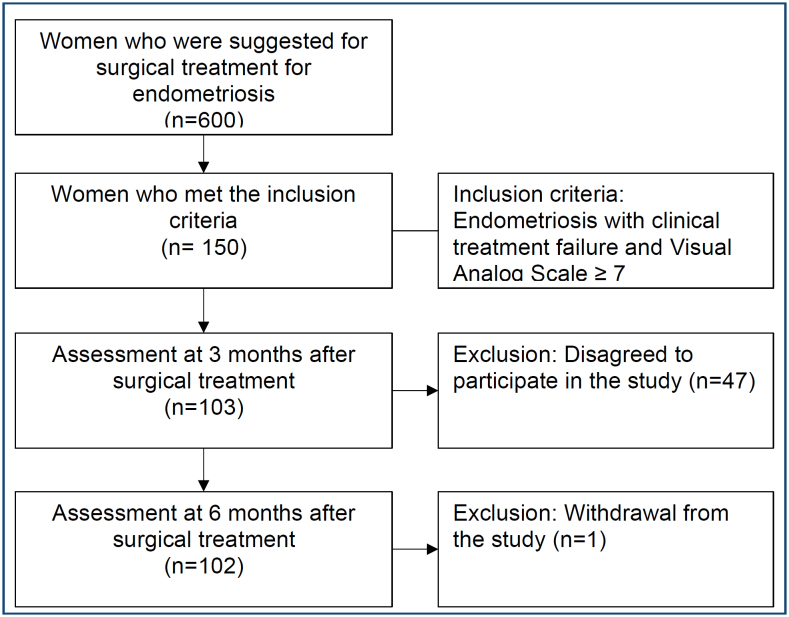
Flowchart of patients at the endometriosis outpatient clinic with indications for surgical treatment for endometriosis from September 2000 to May 2022.

One day before the scheduled date for surgery, the patients responded to the validated Portuguese version of the Beck Depression Inventory II (BDI)^13,14^. The questionnaire was readministered to the patients 3 and 6 months after surgery in a face-to-face format during follow-up appointments. These patients did not undergo any medical treatment for depression during the study (Figure 1).

The BDI assesses the presence of depressive symptoms such as sadness, guilt, past failure, and loss of pleasure, among others^13,14^. The instrument consists of 21 questions, and its results range from 0 to 63. In the validated Portuguese version, which was applied in this study, the intensity of depression is classified based on the score on the inventory as no/minimum depression (0-13), mild (14-19), moderate (20-28) and severe (29-63)^14^. It is important to emphasise that the BDI is not used to diagnose depression but to assess the level of depression.

The patients’ data (age, ethnicity, marital status, if they were overweight, or had had hormone treatments and fertility) were assessed based on medical records. The degree of endometriosis was established intraoperatively according to the modified American Society for Reproductive Medicine (r-ASRM) classification into stages I to IV: minimal, mild, moderate and severe disease^15^. The type of surgery performed was recorded: excision of endometriosis foci, endometriosis+myomectomy, endometriosis+hysterectomy, endometriosis+rectosigmoidectomy, and endometriosis+myomectomy+rectosigmoidectomy and endometriosis+hysterectomy+rectosigmoidectomy. The surgical approach was defined according to the regions affected by the endometriosis foci, the degree of endometriosis involvement and comorbidity with other pelvic pathologies to be operated on.

After collection, the data were processed using the Microsoft Excel 2010 software, in which they were organised into tables and graphs. For statistical analysis, the Statistical Package for Social Sciences (SPSS) version 17.0 was used. Numerical and categorical variables were quantified using absolute and relative frequency measures. The Kolmogorov-Smirnov test was used to assess the normality of the questionnaires. Since the data distribution was not normal, nonparametric data expressed as the median (25th percentile-75th percentile) were used. To compare the different time points, the Friedman test was used, followed by Dunn's post-hoc test.

Data were tabulated in Microsoft Office Excel® (2016 version) (Redmond, WA, USA) and analysed in SPSS (version 21) (Chicago, IL, USA). Data are presented as the mean and standard deviation or median and range (minimum and maximum), and numerical and categorical variables are presented as the absolute number (n) and relative (%) frequency. Normality was assessed using the Shapiro-Wilk test.

To compare the evaluations during follow-up (before, 3 and 6 months after surgery), the Friedman test was applied, with post-hoc analysis using Tukey's test. All statistical associations were set at a significance level of p≤0.05.

The Research Ethics Committee of Hospital São Domingos, São Luís do Maranhão (Brazil), approved and validated the performance of this study through the Brazil Platform. Each participant agreed to participate in the study and completed and signed an informed consent form.

## RESULTS

In total, 600 gynaecological laparoscopic surgeries were performed. A total of 150 patients met the study criteria, of whom 47 did not agree to participate in the study and 103 agreed. One chose not to continue in the study upon return from the second evaluation without informing the reason (Figure 1). All participants had confirmation of endometriosis during the surgical procedure. The final sample consisted of 102 patients, with a mean age of 36±6.3 years. Regarding marital status, 34 (33.3%) were single, and 68 (66.6%) were married. Regarding colour, 18 (17.6%) patients were white, 70 (68.6%) were brown and 14 (13.7%) were black. Regarding body mass index (BMI), 57 (55.8%) were overweight. In clinical treatment prior to surgery, 52 (50.9%) underwent hormonal treatments with progestogens, 30 (29.4%) combined hormonal contraceptives and 21 (20.5%) both. Out of the patients, 40 (39.2%) were infertile and 52 (50.9%) were fertile or did not want to get pregnant. Regarding the degree of the disease, 9 (8.8%) had minimal endometriosis, 18 (17.6%) had mild endometriosis, 35 (34.3%) had moderate endometriosis and 40 (39.2%) had severe endometriosis (Table 1).

**Table 1:** Socio-demographic and clinical characteristics of patients undergoing videolaparoscopy for the treatment of endometriosis.

Variables		n (%)
Age (years) Mean±SD		36±6.3
Colour/race		
	White	18 (17.6)
	Brown	70 (68.6)
	Black	14 (13.7)
Marital status		
	Single	34 (33.3)
	Married	68 (66.7)
	Overweight (BMI³ 25.0 kg/m^2^)	57 (55.8)
Hormone treatments		
	Progestogens	52 (50.9)
	Combined hormonal contraceptives	30 (29.4)
	Both	21 (20.5)
Fertility		
	Infertile	40 (39.2)
	Fertile but did not want to get pregnant	52 (50.9)
Degree of endometriosis (rASRM criteria)		
	Minimum	9 (8.8)
	Lightweight	18 (17.6)
	Moderate	35 (34.3)
	Severe	40 (39.2)
Surgery		
	Endometriosis	21 (20.6)
	Endometriosis + myomectomy	30 (29.4)
	Endometriosis + hysterectomy	16 (15.7)
	Endometriosis + rectosigmoidectomy	20 (19.6)
	Endometriosis + myomectomy + hysterectomy	1 (1.0)
	Endometriosis + myomectomy + rectosigmoidectomy	3 (2.9)
	Others	11 (10.9)

BMI: body mass index; rASRM: revised American Society for Reproductive Medicine; Both: changes the hormone composition due to unwanted effects; Endometriosis: peritoneal, ovarian and deep forms; Others: endometriosis with appendectomy, wall endometriosis, umbilical hernia and intestinal shaving.

The patients underwent the following surgeries: 21 (20.6%) had exeresis of endometriosis foci, 30 (29.4%) had endometriosis+myomectomy, 16 (15.7%) had endometriosis+hysterectomy, 20 (19.6%) had endometriosis+rectosigmoidectomy, 1 (1%) had endometriosis+myomectomy+hysterectomy, 3 (2.9%) had endometriosis+myomectomy+rectosigmoidectomy, and 11 (10.9%) had other types of surgery (Table 1).

There was a reduction in BDI before surgery, from a median of 8 (25th-75th percentile: 3-13) to 2 (0-6) at 3 months after surgery and 0 (0-2) at 6 months after surgery (<0.0001). There was a significant reduction in the BDI mean between 3 and 6 months after surgery, as well as both when compared with the mean before surgery. However, as the mean BDI values in the three groups were within the normal range for the general population, these results should be analysed cautiously (Table 2).

**Table 2:** Comparison between Beck Depression Inventory assessment times for patients undergoing surgical treatment for endometriosis.

Beck Depression Inventory (BDI)	Median (min-max)
T1	8 (0-35)^a^
T2	2 (0-32)^a^
T3	0 (0-27)^a^
p-value £	<0.001

£: Friedman;

a Equal letters indicate a statistically significant difference between the times evaluated.

A reduction was observed in the distribution of women during the study follow-up, where a more significant number of women with moderate or severe symptoms were observed in the first assessment (10.7%) and a lower number in the last assessment (1.0%) (p-value<0.001) (Table 3).

**Table 3:** Comparison between the Beck Depression Inventory categories of patients undergoing videolaparoscopy for the treatment of endometriosis.

BDI categories	BDI T1		BDI T2		BDI T3		p-value
	n	%	n	%	n	%	
Minimal/mild	91	89.3	98	96.0	101	99.0	<0.001¥
Moderate/severe	11	10.7	4	4.0	1	1.0	

BDI: Beck Depression Inventory. ¥ McNemar.

## DISCUSSION

Studies that evaluated women with endometriosis undergoing surgical treatment found that the mean age ranges at the time of surgery were similar to those of our patients^1–16^. Regarding the ethnic profile of the patients in our study, previous studies found a higher prevalence of endometriosis in white patients^1–4^.

The rate of being considered overweight in the female population in general is around 50%, which is compatible with the findings of this research, although studies indicate that being overweight is an indicator of protection against endometriosis^16,17^.

The clinical treatment carried out prior to choosing the study group followed the guidance of the European Society of Human Reproduction and Embryology (ESHRE), where we initially opted for clinical treatment with progestins or oral combined contraceptives, both used continuously, and in cases of undesirable effects, we changed one for the other, forming a third group that used both^1^. The percentage of infertile patients with endometriosis varies in the literature at around 40%, similar to what was observed in the sample of this study^16^.

A meta-analysis on endometriosis and depression showed that patients with chronic pain due to endometriosis have an increased prevalence of depression compared to women with asymptomatic endometriosis^17^. Nevertheless, other factors, such as the possibility of infertility, also influence the association between the two diseases^18^.

There is a possible genetically based aetiological association between depression and endometriosis, as the two conditions share certain gene loci, suggesting a possible direct correlation between depression and endometriosis to some extent^19^. Meta-analyses of genomic association showed that nine reproductive disorders are genetically correlated with each other and are significantly related to perinatal depression, female depression and non-perinatal depression but are related to childbirth and depression in both men and women, with perinatal depression associated with endometriosis^20^. The difference in reproductive hormone levels has been suggested to be the cause of the prevalence of depression, which is more noticeable after puberty, as well as the perinatal period, is affected by hormonal fluctuation and is associated with an increased risk of depression^20^. Depression and anxiety in patients with endometriosis are associated with worse symptoms and a poor prognosis, regardless of pain levels^21^.

Few studies have evaluated depression before and after surgical treatment of endometriosis^22,23^. One study analysed depression in women 2 weeks before and 3 months after undergoing laparoscopic surgery for endometriosis, with a significant reduction in depression^22^. In comparison, our study found lower mean BDI values at all evaluation stages.

Broeck et al. evaluated depression scores in patients surgically treated for endometriosis with and without rectosigmoidectomy and found a significant reduction in the prevalence of moderate or severe depression in both groups before treatment and 18 months after the procedure^23^. In the follow-up after surgery, lower mean values of BDI were achieved among women who had immediate reproductive desire before surgery and actually became pregnant compared to those who had this objective but were unable to become pregnant^23^. In this sample, the follow-up period was longer than that in our study. We should consider that the prevalence of women with severe depressive symptoms was lower before and after surgical treatment in our study. Another point to be considered is that patients with minimal and mild endometriosis were excluded from this study, whereas our study included all stages of endometriosis^23^. In both studies, there was a significant decrease in the BDI scores.

The limiting factors of this study were the size of the selected sample, selection bias, there was no blinding and an excellent team that made generalisation difficult, and the fact that the patients did not receive psychological or psychiatric follow-up during the 6-month study period. However, after the research was completed, all patients were referred for specialised treatment, despite there being a significant reduction in depression with surgical treatment for endometriosis, which mitigates the postponement of specialised follow-up for depression. This may explain the low number of studies with this objective.

## CONCLUSION

The results presented indicate that laparoscopic surgical treatment of endometriosis significantly reduces mild, moderate and severe depressive symptoms, with a possible positive impact on the quality of life of these patients. Long-term studies evaluating the outcome of the procedure with larger samples are needed.
